# Microglia-Dependent and Independent Brain Cytoprotective Effects of Mycophenolate Mofetil During Neuronal Damage

**DOI:** 10.3389/fnagi.2022.863598

**Published:** 2022-04-29

**Authors:** Joshua Kleine, Urszula Hohmann, Tim Hohmann, Chalid Ghadban, Miriam Schmidt, Sebastian Laabs, Beat Alessandri, Faramarz Dehghani

**Affiliations:** ^1^Department of Anatomy and Cell Biology, Medical Faculty, Martin-Luther-University Halle-Wittenberg, Halle (Saale), Germany; ^2^Institute for Neurosurgical Pathophysiology, University Medical Center, Johannes Gutenberg University, Mainz, Germany

**Keywords:** microglia, astrocytes, OHSC, neuroinflammation, IMPDH2, mycophenolate mofetil (MMF), brain cytoprotection

## Abstract

Acute lesions of the central nervous system often lead to permanent limiting deficits. In addition to the initial primary damage, accompanying neuroinflammation is responsible for progression of damage. Mycophenolate mofetil (MMF) as a selective inhibitor of inosine 5-monophosphate dehydrogenase (IMPDH) was shown to modulate the inflammatory response and promote neuronal survival when applied in specific time windows after neuronal injury. The application of brain cytoprotective therapeutics early after neuronal damage is a fundamental requirement for a successful immunomodulation approach. This study was designed to evaluate whether MMF can still mediate brain cytoprotection when applied in predefined short time intervals following CNS injury. Furthermore, the role of microglia and changes in IMPDH2 protein expression were assessed. Organotypic hippocampal slice cultures (OHSC) were used as an *in vitro* model and excitotoxically lesioned with *N*-methyl-aspartate (NMDA). Clodronate (Clo) was used to deplete microglia and analyze MMF mediated microglia independent effects. The temporal expression of IMPDH2 was studied in primary glial cell cultures treated with lipopolysaccharide (LPS). In excitotoxically lesioned OHSC a significant brain cytoprotective effect was observed between 8 and 36 h but not within 8 and 24 h after the NMDA damage. MMF mediated effects were mainly microglia dependent at 24, 36, 48 h after injury. However, further targets like astrocytes seem to be involved in protective effects 72 h post-injury. IMPDH2 expression was detected in primary microglia and astrocyte cell cultures. Our data indicate that MMF treatment in OHSC should still be started no later than 8–12 h after injury and should continue at least until 36 h post-injury. Microglia seem to be an essential mediator of the observed brain cytoprotective effects. However, a microglia-independent effect was also found, indicating involvement of astrocytes.

## Introduction

Acute events like cerebral ischemia or traumatic brain injury (TBI) represent a major challenge in the acute clinical treatment, since lesions of the central nervous system (CNS) are often irreversible and cause severe impairments (Carandang et al., 2006; Maas et al., 2008). In turn, the irreversible primary damage leads to an activation of cellular and biochemical cascades that harm already vulnerable neurons in the perilesional area, inducing and increasing the initial damage (Dirnagl et al., 1999; Jassam et al., 2017). The resulting secondary damage is target of current therapies and the starting point for new treatment approaches (Hailer, 2008; Chamorro et al., 2016; Nogueira et al., 2018). The progression of secondary damage is characterized by the inflammatory response, which sets the level of further specific immunological reactions (Chamorro et al., 2012; Jassam et al., 2017). At cellular level, cell-cell interactions determine the responses between microglia, neurons, astrocytes, endothelial cells, and infiltrated peripheral immune cells (Nedergaard and Dirnagl, 2005; Ahmad et al., 2013; Burda and Sofroniew, 2014). In addition, different soluble factors such as cytokines, chemokines, and reactive oxygen species seem to play an essential role in secondary damage (Hailer, 2008; Dirnagl and Endres, 2014). The reduction of inflammation and secretion of proinflammatory factors by immunosuppressive agents is believed to be an effective strategy. Mycophenolate mofetil (MMF) was first used for immunosuppression after kidney transplantations (Allison et al., 1993). After administration, MMF is rapidly hydrolyzed to its active form of mycophenolic acid (Lee et al., 1990) and inhibits both isoforms of inosine 5-monophosphate dehydrogenase (IMPDH), the rate limiting enzyme in the *de novo* synthesis of guanosine nucleotides. MMF therefore counts as a noncompetitive and reversible inhibitor with a 5-fold higher affinity to IMPDH2 than the type 1 isoform. Whereas IMPDH2 was induced predominantly in activated leukocytes and organotypic hippocampal slice cultures (OHSC), the type 1 isoform was constitutively expressed in most cell types (Allison and Eugui, 2000; Gu et al., 2003; Ebrahimi et al., 2012). MMF application resulted in specific inhibition of proliferating leukocytes *via* IMPDH2 (Jonsson and Carlsten, 2002). In astrocytes and microglia, MMF inhibited the proliferation and reduced the release of inflammatory cytokines like IL 1ß, TNF α, and NO (Miljkovic et al., 2002; Dehghani et al., 2010). In accordance, treatment with MMF in excitotoxically damaged OHSC showed a brain cytoprotective effect in addition to a reduction in glia proliferation (Dehghani et al., 2003; Ebrahimi et al., 2012) and increased survival of long range axonal projections (Oest et al., 2006). Direct effects on neuronal cells were not described since MMF failed to affect neuronal survival in NMDA damaged primary neuronal cell cultures (Dehghani et al., 2010). Thus, MMF probably affects the glial cells directly or rather the interactions between glial cells and neurons. Interestingly, MMF mediated effects showed a temporal pattern since MMF was protective after lesion in a specific time window of 12–36 h in OHSC and significantly reduced the extent of neuronal damage when administered continuously in the first 12 h after injury (Ebrahimi et al., 2012). MMF mediated brain cytoprotection was abolished when the treatment was started later, between 24 and 48 h (Ebrahimi et al., 2012).

In accordance to the Stroke Therapy Academic Industry Roundtable (STAIR) recommendations, the evaluation of brain cytoprotection took place in different time scales for MMF treatment in OHSC (Lyden et al., 2021). Furthermore, microglia independent effects were investigated and the expression dynamics of IMPDH2 in primary microglia and astrocytes after lipopolysaccharide (LPS) stimulation analyzed.

## Materials and Methods

### Ethics Statement

All animal experiments were performed in accordance with the Policy on Ethics and the Policy on the Use of Animals in Neuroscience Research as indicated in the directive 2010/63/EU of the European Parliament and of the Council of the European Union on the protection of animals used for scientific purposes and were approved by the local authorities for care and use of laboratory animals (State of Saxony-Anhalt, Germany, permission number: I11M18, date: 01.12.2012).

### Organotypic Hippocampal Slice Cultures

Organotypic hippocampal slice cultures were prepared under aseptic conditions from 8 to 9 days old Wistar rats as described before (Grabiec et al., 2017). Briefly, the frontal pole and the cerebellum were removed and the brains were placed in preparation medium, consisting of minimum essential medium (Invitrogen) and 1% (w/v) L-glutamine (Invitrogen), at 4°C and pH 7.35. Brains were sliced horizontally into 350 μm thick sections (Vibratome VT 1200 S; Leica Microsystem, Wetzlar, Germany). Approximately six to eight OHSC were obtained from each brain. After preparation, slices were transferred into culture inserts (Sarstedt, Nümbrecht, Germany) and incubated with 1 ml of culture medium. The medium consisted of 50 ml minimum essential medium (Invitrogen), 25 ml Hank’s balanced salt solution (HBSS, Thermo Fisher), 25 ml heat inactivated horse serum (Invitrogen), 2 ml L-glutamine (Invitrogen), 1 μg/ml insulin (Boehringer, Mannheim, Germany), 1.2 mg/ml glucose (Merck Millipore), 0.1 mg/ml streptomycin and penicillin and 0.8 μg/ml vitamin C (Sigma) at pH 7.4. OHSC were incubated for 6 days at 35°C with 5% CO_2_ and the culture medium was changed every second day.

**FIGURE 1:**
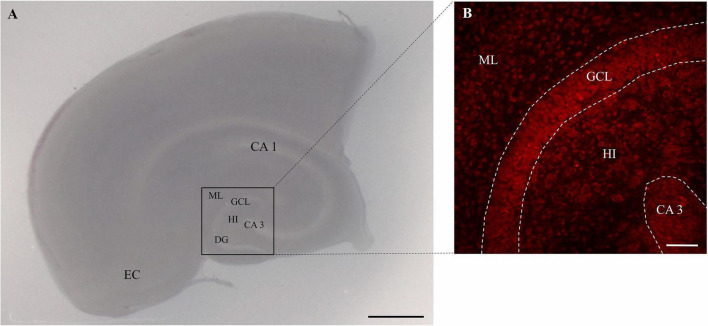
Image of rat OHSC. **(A)** OHSC in culture with intact cytoarchitecture. The entorhinal cortex (EC), cornu ammonis (CA) subfields CA 1 and CA 3, the hilus region (HI) and the dentate gyrus (DG) with the molecular layer (ML) and the granule cell layer (GCL) are highlighted. Scale bar = 1 mm. Panel **(B)** represents the region of interest in the OHSC. The morphology is visualized by the use of PI staining (red). The dotted areas show the GCL and the CA3 regions. Scale bar = 50 μm.

After 6 days OHSC treatment with 50 μM N-methyl-D-aspartate (NMDA, Merck) was performed for 4 h. The OHSC (Figure 1) were randomly assigned to the different treatment groups and treated according to the respective protocols. Microglia were depleted from OHSC using the bisphosphonate clodronate (Clo, Bonefos^®^, Bayer Vital GmbH, Leverkusen, Germany). Sections were incubated with 100 μg/ml Clo in the respective treatment groups for 6 days starting directly after preparation. At the end of experiments the sections were rinsed with 0.1 M phosphate buffer (PB) and then fixed with 4% (w/v) paraformaldehyde (PFA, AppliChem, Darmstadt, Germany) and stored at 4°C.

#### Control

**FIGURE 2:**
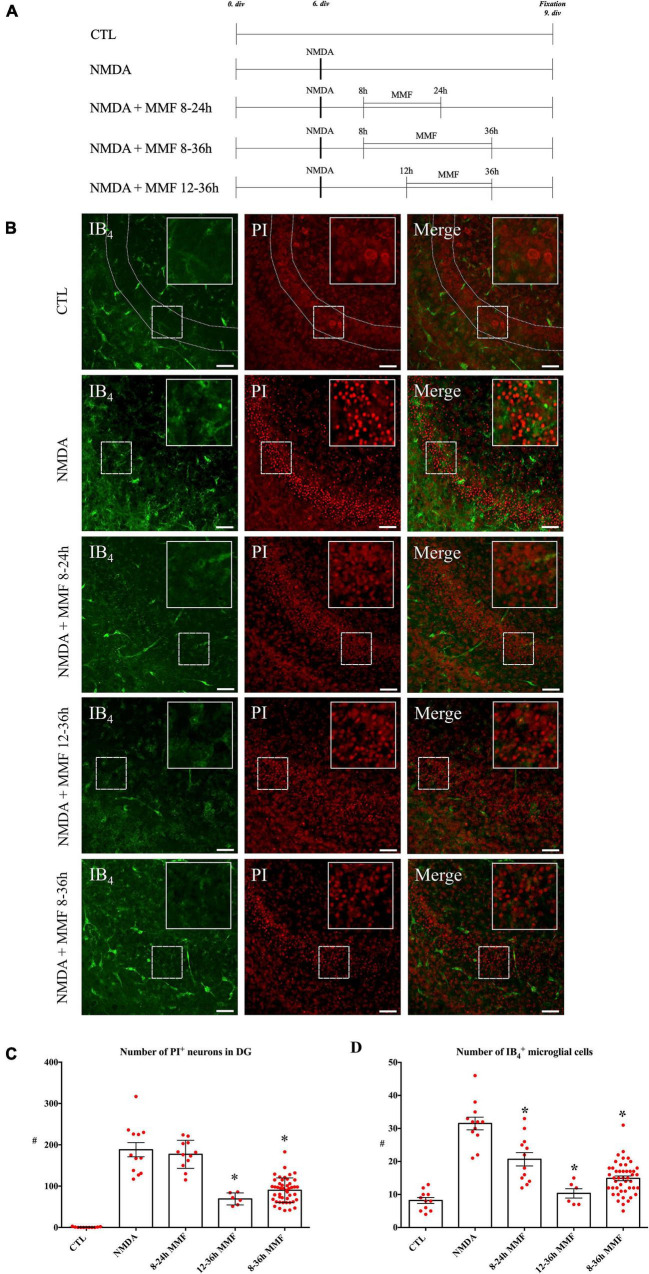
Effects of MMF application in a specific time window in NMDA damaged OHSC fixed at 9 div. **(A)** Treatment protocol. **(B)** CLSM images stained with PI (degenerating neurons, red) and IB_4_ (microglial and vessels, green) in overview and in higher magnification of the labeled area. Control (CTL, *n* = 5) OHSC showed a well preservation of the hippocampal formation with almost no PI positive pyknotic nuclei and only a few ramified IB_4_ positive microglia. OHSC treated with NMDA (*n* = 13) (50 μM) for 4 h had massive accumulation of PI positive degenerating neurons and amoeboid IB_4_ positive microglia. Treatment with MMF (100 μg/ml) in defined time windows resulted in a reduction of PI positive neurons between 12 and 36 h (*n* = 6) and 8 and 36 h (*n* = 43) but not between 8 and 24 h (*n* = 12) after NMDA damage. In all time windows, there was a reduction in the number of microglia. Quantitative analyses of the mean numbers of panel **(C)** PI positive degenerating neurons (*p < 0.05 vs. NMDA) and **(D)** IB_4_ positive microglia (*p < 0.05 vs. NMDA). The asterisk denotes significant results regarding the respective measurement indicated with the bar. The values are served as a mean with standard error of the mean. Scale bars = 50 μm.

**FIGURE 3:**
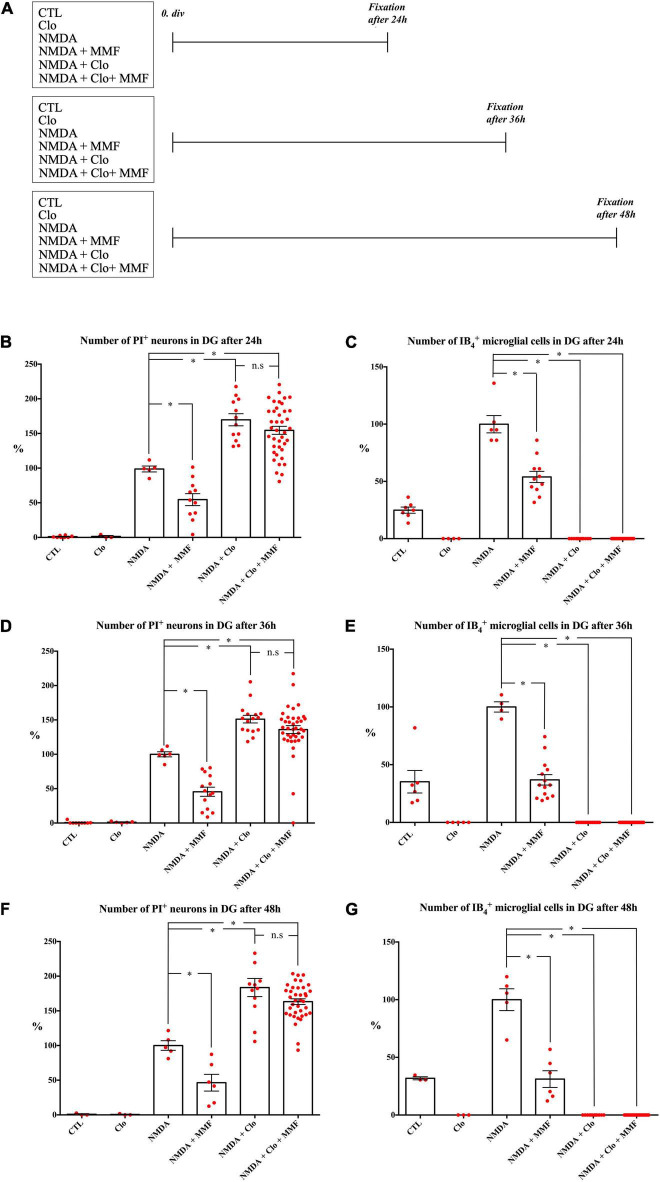
Effects of continuous treatment with MMF for OHSC fixed at 24, 36, and 48 h after injury with and without (Clo) microglia. **(A)** Treatment protocol. The slices were fixed 24, 36, or 48 h after NMDA damage. Quantitative analysis of PI positive degenerating neurons after **(B)** 24 h, **(D)** 36 h, and **(F)** 48 h and microglia after **(C)** 24 h, **(E)** 36 h, and **(G)** 48 h. The asterisk denotes significant results regarding the respective measurement indicated with the bar (*p < 0.05 vs. NMDA, n.s. *p* > 0.05 vs. NMDA + Clo). The values are served as a mean with standard error of the mean.

**FIGURE 4:**
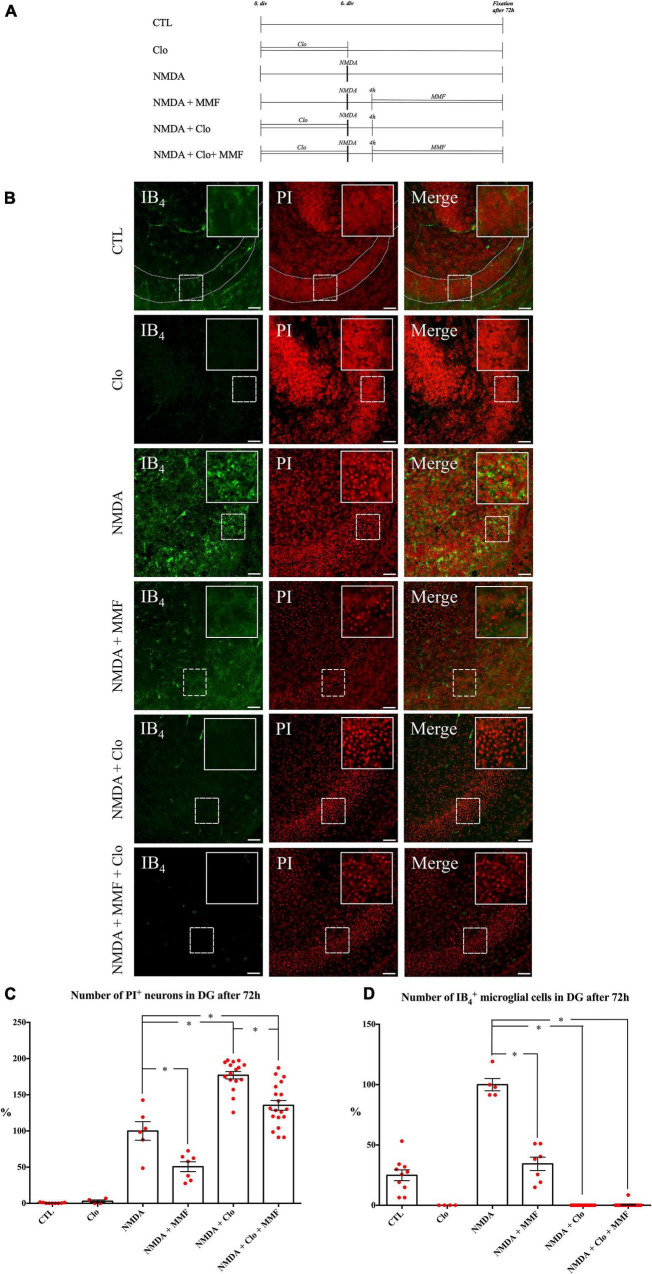
Effects of continuous MMF treatment for OHSC fixed at 72 h after injury with and without microglia **(A)** Treatment protocol. **(B)** CLSM images stained with PI (degenerating neurons, red) and IB_4_ (microglial cells vascular vessels, green) in overview and in higher magnification of the labeled area. In comparison to control (CTL, *n* = 9), treatment with NMDA for 4 h (NMDA, *n* = 6) led increase in number of PI positive degenerating neurons. Treatment with MMF (NMDA + MMF, *n* = 7) in a period between 4 and 72 h after the injury resulted in a reduction of PI positive degenerated neurons. Incubation with 100 μg/ml Clo for 6 days resulted in successful depletion of microglia from OHSC in the respective groups (Clo, *n* = 4; NMDA + Clo, *n* = 16; NMDA + Clo + MMF, *n* = 19). Additional application of MMF in this period led to reduction of PI positive degenerated neurons in microglia depleted OHSC (NMDA + Clo + MMF). Depletion of microglia led to an increase in number of damaged cells (NMDA + Clo, NMDA + Clo + MMF). Quantitative analyses of the mean numbers of panel **(C)** PI positive degenerating neurons (*p < 0.05 vs. NMDA or NMDA + Clo) and **(D)** IB_4_ positive microglia (*p < 0.05 vs. NMDA). The asterisk denotes significant results regarding the respective measurement indicated with the bar. The values are served as a mean with standard error of the mean. Scale bars = 50 μm.

Control OHSC (*n* = 34) were treated with culture medium only till fixation (Figures 2A, 3A, 4A).

#### *N*-Methyl-Aspartate

On day 6 OHSC (*n* = 35) were lesioned with NMDA (50 μM; Sigma Aldrich) for 4 h and were further incubated with culture medium until the time of fixation (Figures 2A, 3A, 4A). The same treatment was done for all NMDA damaged groups.

#### Mycophenolate Mofetil Time Window

Different concentrations of MMF were tested before (Dehghani et al., 2003; Ebrahimi et al., 2012). To analyze the brain cytoprotective time window, damaged OHSC were treated with MMF (100 μg/ml) at different time intervals. MMF was applied between 8–24 h (*n* = 12), 8–36 h (*n* = 43), and 12–36 h (*n* = 6) after starting with NMDA treatment (Figure 2A).

#### Clodronate

Microglia depleted OHSC were supplied with culture medium. Fixation was performed after 24 h (*n* = 3), 36 h (*n* = 5), 48 h (*n* = 3), and 72 h (*n* = 4) (Figures 3A, 4A). The same treatment was conducted with 100 μg/ml Clo for all Clo treated groups.

#### Continuous Mycophenolate Mofetil Treatment in *N*-Methyl-Aspartate Lesioned Organotypic Hippocampal Slice Cultures

Treatment with MMF (100 μg/ml) to NMDA damaged OHSC was performed on day 6. Fixation was carried out after 24 h (*n* = 11), 36 h (*n* = 14), 48 h (*n* = 6), and 72 h (*n* = 7) (Figures 3A, 4A).

#### *N*-Methyl-Aspartate Mediated Damage in Microglia Depleted Organotypic Hippocampal Slice Cultures

Fixation was performed after 24 h (*n* = 12), 36 h (*n* = 16), 48 h (*n* = 12) and 72 h (*n* = 16) after NMDA damage (Figures 3A, 4A).

#### Continuous Treatment With Mycophenolate Mofetil in *N*-Methyl-Aspartate Damaged Microglia Depleted Organotypic Hippocampal Slice Cultures

*N*-methyl-aspartate treatment was followed by application of MMF (100 μg/ml). Fixation was performed after 24 h (*n* = 40), 36 h (*n* = 39), 48 h (*n* = 38), and 72 h (*n* = 19) (Figures 3A, 4A).

### Primary Cell Cultures

Primary microglia and astrocytes were isolated from cerebral cortices of neonatal wild type rats as described before (Dehghani et al., 2003). Briefly, brains were treated with 0.5 mg/ml DNAse (Worthington, Bedford, MA, United States) and 4 mg/ml trypsin (Merck Millipore, Burlington, MA, United States) in Hank’s balanced salt solution (Invitrogen). Primary cells were cultured in DMEM (Invitrogen) with 10% (w/v) FBS (Invitrogen) and 1 ml streptomycin/penicillin (Invitrogen) as described before (Grabiec et al., 2012). Microglia or astrocytes were seeded into 6 well plates (250,000 cells/well) and incubated in DMEM (Invitrogen) with 2% (w/v) fetal bovine serum for 24 h prior to treatment. Depending on the group, the cells were treated with lipopolysaccharide (LPS, 10 ng/ml, Sigma Aldrich, Deisenhofen, Germany) or left untreated (CTL).

### Western Blot Analyses

For western blot analyses cells were collected 0, 6, 8, 12, 16, and 24 h after treatment, respectively. The cells were taken up in lysis buffer and stored at −80°C as described before (Grabiec et al., 2019). Protein concentrations were determined using the method of Bradford (Pierce™ BCA Protein Assay Kit, Thermo Fisher Scientific, Barrington IL, United States) (Bradford, 1976) and 10 μg protein was loaded onto a 12.5% (w/v) sodium dodecyl sulfate–polyacrylamide gel. After gel electrophoresis, the proteins were blotted onto nitrocellulose membranes (Protran 0.45 μm, Amersham, Freiburg, Germany) and non-specific protein binding sites were blocked for 30 min with roti block solution (Carl Roth, Karlsruhe, Germany). The nitrocellulose membranes were incubated with the antibody against IMPDH2 (Table 1) [diluted 1:2000 in roti block solution containing 0.2% (v/v) Tween 20] until the next day at 4°C. After incubation with horseradish peroxidase conjugated antibodies (Table 1) the chemiluminescent (Luminata Forte, Merck) signal was detected with Fusion X (VWR, Radnor, PA, United States). For semi quantitative analysis, the relative signal intensities of the immunoreactive bands were determined and the IMPDH2 intensity was normalized to the ß-actin (Table 1) intensity. Fusion FX7 with FusionCapt Advance Solo software (VWR) was used for analysis.

**TABLE 1:** Antibodies: western blot analysis.

Name	Company	Number	Dilution	Antibody ID
IMPDH 2	Proteintech Group, Rosemont, IL, United States	12948-1-AP	1:2000	
Goat anti-rabbit IgG, HRP conjugated	Vector Laboratories	PI-1000	1:20000	AB_2336198
Horse anti-mouse IgG, HRP conjugated	Vector Laboratories	PI-2000	1:10000	AB_2336177
ß-actin	Cell Signaling, Boston, United States	3700	1:5000	AB_2242334

### Immunofluorescence, Microscopy, and Image Acquisition

To visualize neuronal cell death OHSC were stained with 5 μg/ml of propidium iodide (Figure 1B; PI, Merck) 2 h before fixation. Microglia were labeled with fluorescein isothiocyanate (FICT)-conjugated isolectin B_4_ (IB_4_, Vector Laboratories, Burlingame, CA, United States) diluted 1:50 in PBS/Triton for 24 h. Before staining, the OHSC were incubated in normal goat serum (NGS, Sigma Aldrich, 1:20) in PB (0.2 M) for 30 min. Thereafter the sections were washed with PBS/Triton and water before the OHSC were covered with the DAKO fluorescence medium (DAKO, Hamburg, Germany). The OHSC were imaged with a confocal laser scanning microscope (LSM 710 Meta, Carl Zeiss AG, Göttingen, Germany). PI was visualized with monochromatic light at 543 nm and an emission bandpass filter of 585–615 nm. IB_4_ was detected with monochromatic light at 488 nm and an emission bandpass filter of 505 to 530 nm. The images were taken using a z-stack with a section distance of 2 μm. On average, 13–17 optical sections were obtained from each OHSC. For PI and IB_4_ analyses, only the three middle sections of an OHSC were evaluated at 200-fold magnification with a resolution of 1024×1024 pixels. The number of PI and IB_4_ positive cells was determined by the use of self-written script in MATLAB (MathWorks, Natick, MA, United States).

### Statistical Analysis

Statistical analysis was performed with Prism 6 (GraphPad Software, San Diego, CA, United States). The analysis for the normal distribution was performed using D’Agnostino and Pearson and Shapiro–Wilk normality test. For statistical analysis, Mann Whitney test was performed as a non-parametric test. Further, the one-way ANOVA was used followed by Bonferroni’s test for multiple comparisons between the groups which yielded identical results. The data were presented as mean and SEM. Differences were considered significant at *p* ≤ 0.05. All experiments were carried out in at least three independent biological replicates.

## Results

### Mycophenolate Mofetil Mediated Effects in Organotypic Hippocampal Slice Cultures Begin Within 8 h of Injury and Last Over 24 h

Control OHSC showed a well-preserved cytoarchitecture, and only very few PI positive cells were observed (Figures 2A–C, CTL: 0.64 PI positive cells/GCL). Microglia showed a ramified morphology and were located mostly in the molecular layer of the DG (Figures 2B,D, CTL: 8.18 IB_4_ positive cells/GCL). NMDA treatment led to a massive accumulation of PI positive neurons in DG (Figures 2B,C, NMDA: 188.1 PI positive cells/GCL), and the number of IB_4_ positive microglia increased significantly (Figures 2B,D, NMDA: 31.5 IB_4_ positive cells/GCL).

When compared to the NMDA group, MMF treatment from 8 h to 24 h (NMDA + MMF 8–24 h: 177.0 PI positive cells/GCL) showed no significant reduction in the number of PI positive neurons (Figures 2B,C) but significantly reduced the number of microglia (Figures 2B,D, 22.9 IB_4_ positive cells/GCL vs. NMDA, *p* < 0.01). MMF treatment from 12–36 h (NMDA + MMF 12–36 h: 69.0 PI positive cells/GCL vs. NMDA, *p* < 0.001) post-injury showed a significant reduction of PI positive neurons (Figures 2B,C) and microglia (Figures 2B,D, 11.5 IB_4_ positive cells/GCL vs. NMDA *p* < 0.0001). Similarly, MMF application in the time-window of 8–36 h (NMDA + MMF 8–36 h: 90.1 PI positive cells/GCL vs. NMDA, *p* < 0.0001) significantly reduced the number of PI positive cells (Figures 2B,C) and microglia when compared to the NMDA group (Figures 2B,D, NMDA + MMF 8–36 h: 14.9 IB_4_ positive cells/GCL vs. NMDA, *p* < 0.001). There was no significant difference in the number of PI positive neurons or IB_4_ positive microglia between the time windows 8–36 h and 12–36 h (Figures 2C,D, *p* > 0.05).

### Consistent Microglia Dependent Brain Cytoprotective Effects of Mycophenolate Mofetil at 24, 36, 48, and 72 h After Injury

To study the temporal development of the brain cytoprotective effect of MMF, the extent of the damage and the number of microglia were analyzed at different fixation times (Figures 3, 4), namely after 24 h (Figures 3A–C), 36 h (Figures 3A,D,E), 48 h (Figures 3A,F,G), and 72 h (Figures 4B–D). Across the different time points, all OHSC controls (CTL) showed a well-preserved cytoarchitecture in DG with only a few isolated PI positive cells and indifferent ramified morphology of the microglia (Figure 4, Supplementary Figures 1–3, and Table 2). After injury with NMDA, a significant accumulation of PI positive neurons in DG, particularly after 24, 36, 48, and 72 h, was observed in all NMDA treated groups (Figures 3B,D,F, 4C and Table 2). Additionally, a significant increase in the number of IB_4_ positive microglia was observed at all time points, with a peak after 36 h (Figures 3C,E,G, 4D, NMDA, Supplementary Figure 4, and Table 2). Treatment with MMF (NMDA + MMF) after 24 h showed a significant reduction in degenerated PI positive neuronal cells/GCL, that remained stable after 36, 48, and 72 h (Figures 3, 4, Supplementary Figures 1–3, and Table 2). The number of IB_4_ positive microglia was also significantly reduced at all time points (Figures 3, 4, NMDA + MMF and Table 2). Although 24 h after MMF treatment, the number of microglia was slightly higher than in the OHSC controls (Supplementary Figure 4). Already after 36 h as well as at the subsequent time points, an adaptation to the control level in the number of microglia was observed (Figures 3C,E,G, 4B,D, Supplementary Figure 4, NMDA + MMF, and Table 2). In an analogous manner, there were already fewer amoeboid microglia, partially also with ramified morphology, after 24 h consistently over the further time points (Supplementary Figures 1–3). There was a significant strong correlation between the number of PI and IB_4_ positive cells (correlation coefficient 219 = 0.631 [0.485, 0.743], Supplementary Figure 5).

**TABLE 2:** Brain cytoprotective effects of continuous MMF treatment at different points in time with or without depletion of microglia.

Treatment	n	Standard deviation PI	PI no. (%)	Mean Diff. % (95% CI)†, ‡. *,**	Standard deviation IB_4_	IB_4_ no. (%)	Mean Diff. % (95% CI)†,*,**
**24 h**							
CTL	8	1.42	3.6 (1.1)	*p* = 0.0016* 98.9 (50.98–144.30)†	7.15	13.6 (24.9)	*p* = 0.0012* 75.0 (63.30–86.77)†
Clo	3	2.42	4.0 (1.4)	*p* = 0.0357* 98.6 (37.56–157.10)†	0.0	0.0 (0.0)	*p* = 0.0095* 100 (86.31–113.54)†
NMDA	5	9.57	286.2 (100)		18.57	44.2 (100)	
NMDA + MMF	11	28.72	160.7 (54.5)	*p* = 0.0087* 45.5 (0.39–88.05)†	16.15	23.8 (53.9)	*p* = 0.0004* 46.9 (35.33–56.73)†
NMDA + Clo	12	30.13	485.8 (169.8)	*p* = 0.0003* 69.8 (27.76–114.30)†	0.0	0.0 (0.0)	*p* < 0.0001* 100 (89.38–110.47)†
NMDA + Clo + MMF	40	36.27	442.2 (154.5)	*p* = 0.0008* 54.5 (17.23–94.32) 15.2 (−7.91 to 38.39)‡ *p* = 0.2167**	0.0	0.0 (0.0)	*p* < 0.0001* 100 (90.68–109.17) *p* > 0.9999**
**36 h**							
CTL	9	1.736	3.2 (0.7)	*p* = 0.0002* 99.3 (60.76–138.00)†	23.87	18.5 (35.2)	*p* = 0.0095* 64.8 (48.77–80.76)†
Clo	5	1.066	5.0 (1.3)	*p* = 0.0043* 98.7 (54.38–143.10)†	0.0	0.0 (0.0)	*p* = 0.0079* 100 (83.38–116.62)†
NMDA	6	9.088	286.1 (100)		8.87	52.5 (100)	
NMDA + MMF	14	24.72	130.1 (45.5)	*p* < 0.0001* 54.5 (18.77–90.26)†	16.85	19.4 (36.9)	*p* = 0.0007* 63.13 (49.08–77.18)†
NMDA + Clo	16	22.14	432.5 (151.1)	*p* < 0.0001* 51.1 (16.07–89.19)†	0.0	0.0 (0.0)	*p* = 0.0002* 100 (86.15–113.85)†
NMDA + Clo + MMF	39	35.66	399.2 (135.9)	*p* = 0.0002* 35.9 (3.80–68.04) 15.2 (−4.09 to 34.51)‡ *p* = 0.0892**	0.0	0.0 (0.0)	*p* < 0.0001* 100 (86.98–113.02) *p* > 0.9999**
**48 h**							
CTL	3	1.841	2.0 (1.3)	*p* = 0.0357* 98.7 (32.9–164.47)†	2.35	16 (32.5)	*p* = 0.0357* 67.5 (50.62–84.33)†
Clo	3	1.228	5.4 (0.9)	*p* = 0.0357* 99.1 (33.36–164.91)†	0.0	0.0 (0.0)	*p* = 0.0357* 100 (83.14–116.86)†
NMDA	5	15.45	230.4 (100)		21.19	49.2 (100)	
NMDA + MMF	6	29.52	107.0 (46.5)	*p* = 0.0087* 53.56 (5.95–101.16)†	17.75	15.3 (31.2)	*p* = 0.0043* 68.84 (56.63–81.03)†
NMDA + Clo	12	45.18	423.1 (183.6)	*p* = 0.0023* 83.6 (41.78–125.48)†	0.0	0.0 (0.0)	*p* = 0.0002* 100 (89.28–110.72)†
NMDA + Clo + MMF	38	25.30	376.1 (163.3)	*p* < 0.0001* 63.3 (25.85–100.65) 20.4 (−0.27 to 41.02)‡ *p* = 0.0714**	0.0	0.0 (0.0)	*p* < 0.0001* 100 (90.42–109.58) *p* > 0.9999**
**72 h**							
CTL	9	0.6651	1.2 (0.5)	*p* = 0.0004* 99.5 (67.34–131.70)†	14.08	11.7 (24.9)	*p* = 0.0007* 75.1 (63.06–87.16)†
Clo	4	3.491	7.3 (2.9)	*p* = 0.0095* 97.1 (57.67–136.48)†	0.0	0.0 (0.0)	*p* = 0.0079* 100 (85.24–114.76)†
NMDA	6	31.56	246.8 (100)		11.36	47 (100)	
NMDA + MMF	7	17.86	125.0 (50.6)	*p* = 0.014* 49.4 (15.40–83.33)†	14.71	16.1 (34.3)	*p* = 0.0025* 65.7 (52.77–78.54)†
NMDA + Clo	16	20.51	436.9 (177.0)	*p* < 0.0001* 77.0 (47.78–106.23)†	0.0	0.0 (0.0)	*p* < 0.0001* 100 (88.64–111.36)†
NMDA + Clo + MMF	19	29.39	334.2 (135.4)	*p* = 0.0258* 35.4 (6.79–63.97) 41.6 (23.85–59.38)‡ *p* < 0.0001**	1.95	0.0 (0.0)	*p* < 0.0001* 100 (88.493–110.61) *p* > 0.9999**

Sample size, standard deviation, and mean values for all treatments. *Mann-Whitney Test vs. NMDA. †One-way ANOVA vs. NMDA. ‡t-test vs. NMDA + Clo. **Mann-Whitney Test vs. NMDA + Clo.

### Mycophenolate Mofetil Showed Microglia-Independent Brain Cytoprotective Effects

In addition, the effects of MMF were assessed at the same time points after depletion of microglia by clodronate (Clo). Treatment with Clo only led to analogous results on PI positive cells as obtained for CTL across all time points. Additionally, a complete reduction was observed in the number of microglia (Figures 3C,E,G, 4B,D, Supplementary Figures 1–3, and Table 2). Treatment with NMDA in OHSC after microglia depletion led to a significant increase in neuronal damage (Table 2) that strongly exceeded the injury as observed in the presence of microglia. This observation was consistent for all analyzed time points (Figures 3, 4 and Table 2). Thereby, the maximum was already reached after 24 h (Figures 3B,C, Supplementary Figure 4, and Table 2). After additional treatment with MMF, no significant differences were found in comparison to the NMDA + Clo group after 24 h (Figure 3C), 36 h (Figure 3E), and 48 h (Figure 3G). However, 72 h after the injury a significant reduced number of PI positive degenerated neurons was detected in the DG (Figures 4B,C and Table 2).

### Inosine 5-Monophosphate Dehydrogenase 2 Expression in Primary Microglia and Astrocytes

**FIGURE 5:**
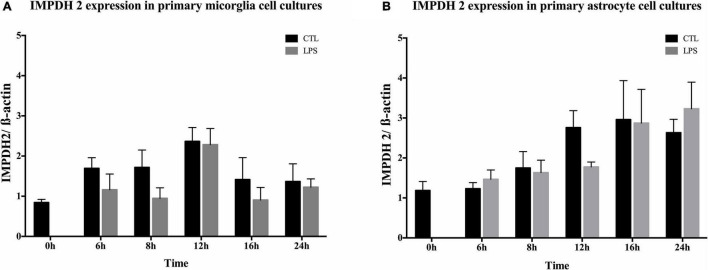
Inosine 5-monophosphate dehydrogenase 2 (IMPDH2) expression in primary microglia and astrocytes. Quantitative analysis of IMPDH2 and ß-actin expression in primary microglia **(A)** and astrocytes **(B)** over time (*n* = 3). IMPDH2 was present at all times in both primary cell lines. Treatment of primary microglia and astrocytes with LPS (10 ng/ml) had no significant effect (*p* > 0.05) on the expression.

The expression of IMPDH2 was investigated in primary microglia (Figure 5A) or astrocyte (Figure 5B) cell cultures at different time points. IMPDH2 was detected with a single band at 56 kDa after 6, 8, 12, and 24 h (Supplementary Figure 6). In relation to ß-actin, no changes were observed after stimulation with LPS for all examined time points in microglia and astrocytes (*p* > 0.05, Figures 5A,B). Furthermore, a LPS independent increase in IMPDH2 expression was evident in the primary microglia cell cultures after approximately 12 h (Figure 5A) and in the primary astrocyte cultures after 16 and 24 h (Figure 5B).

## Discussion

Acute damage to the CNS, such as TBI or stroke, often leads to permanent limiting deficits (Carandang et al., 2006; Feigin et al., 2014). Whereas primary damage is the result of acute and usually immediate injury, secondary damage progresses over hours and days and is a consequence of various complex processes, including excitotoxicity, overproduction of free radicals, and inflammation (Leker et al., 2002; Werner and Engelhard, 2007). During secondary damage, microglia and astrocytes act as important cellular players and contribute to the neuroinflammatory response (Morshead and van der Kooy, 1990). However, depending on its severity, the inflammation might lead to further damage of already vulnerable neurons, but could also impact functions that are important for their survival (Dirnagl et al., 1999; Ebrahimi et al., 2010). A promising way for therapeutic intervention might be the blockade of defined intrinsic immunological actions after injury (Chamorro et al., 2016). Immunosuppression has nonetheless some disadvantages, like increased risk of infection (Husain and Singh, 2002). For this reason, it is crucial to limit the interventional time to minimize its side effects.

### Modulation of Neuroinflammation as a Brain Cytoprotective Approach

Treatment with the immunosuppressant MMF, a competitive inhibitor of key enzyme in purine metabolism namely IMPDH2, was reported to significantly reduce the neuronal cell death after excitotoxic lesions in OHSC (Dehghani et al., 2003). OHSC represent an established model for intrinsic study of glia cells, neurons and their interactions without the influence of peripheral migrating immune cells or blood flow (Grabiec et al., 2017). A temporal relationship was previously described for MMF resulting in brain cytoprotective effects, saving further cell types and the structure in OHSC (Ebrahimi et al., 2012). A brain cytoprotective time window was found for MMF between 12 and 36 h after injury. However, due to negative effects of immunosuppression, the onset and necessary duration of MMF treatment needed to be further evaluated. Furthermore, it is assumed that the earlier an effective protection is started, the better the neuronal survival. This raised the question whether an earlier start and timely limited treatment may also be protective. Especially in the context of stroke, infections are a feared complication partly due to the so-called stroke-induced immunodepression (Klehmet et al., 2009; Hoffmann et al., 2017). Therefore, the application of therapeutics with immune interfering capabilities should occur as early as possible and for a limited period of time. In addition, new interventional procedures such as thrombectomy led to ever wider clinical therapeutic time windows beyond the 6 h for initiation of therapeutic measures (Nogueira et al., 2018). Therefore, the establishment of temporally appropriate brain cytoprotective approaches is becoming increasingly important.

Recent studies involving immunosuppressive agents indicate brain cytoprotective effects by influencing glia cells function similar to MMF (Zawadzka and Kaminska, 2005; Erlich et al., 2007). Treatment with immunosuppressant FK506 significantly reduced the lesion volume in induced cerebral infarction (Sharkey and Butcher, 1994; Zawadzka and Kaminska, 2005) and affected the activity of microglia as well as astrocyte and cytokine production *in vitro* and *in vivo* (Muramoto et al., 2003; Zawadzka and Kaminska, 2005). These findings are in accordance with MMF data, showing the modulating action of microglia, astrocytes, and cytokine production (Dehghani et al., 2010). Brain cytoprotective effect of immunosuppressant FK506 was also demonstrated in OHSC (Lee et al., 2010). Roscovitine, an inhibitor of CDK5 with additional immunosuppressive effects reduced neuronal loss, glial activation, as well as microglia proliferation, NO release, and neurologic deficits after brain trauma or stroke (Di Giovanni et al., 2005; Hilton et al., 2008; Menn et al., 2010). Treatment with rapamycin, a further immunosuppressant, increased neural survival thus improving the functional recovery and reduced the number of microglia/macrophages after TBI (Erlich et al., 2007; Lipton and Sahin, 2014). In most cases, the substances in the studies were administered in a very early period after injury, mostly within the first few hours. Further temporal aspects of the application were not considered, although it is necessary for the clinical practice. In a several studies, a specific brain cytoprotective time window was demonstrated. For example, FK506 showed a brain cytoprotective time window *in vivo* within the first 120 min but not if applied later in an occlusion model (Arii et al., 2001; Furuichi et al., 2003). A better characterization of the so called windows of opportunity is needed *in vitro* and *in vivo*. The aspect of a therapeutic time-defined approach gains more and more importance due to the necessity of standardized preclinical study settings (Feuerstein et al., 2008; Lyden et al., 2021).

### The Brain Cytoprotective Effect of Mycophenolate Mofetil Depends on the Time of Application

In our previous work, MMF showed a protective time window of 12–36 h in OHSC (Ebrahimi et al., 2012). Continuous treatment up to 12 h after the injury led to a reduction in a number of damaged neurons and microglia. Later onset of MMF treatment was no longer linked to brain cytoprotective effects. While treatment between 24–48 h after injury failed to demonstrate any benefits, a brain cytoprotective effect was identified for treatment duration of up to 12 and 36 h (Ebrahimi et al., 2012). In this regard, the investigation of an earlier start of MMF treatment in the context of new clinical therapeutic interventions and a lower risk of systematic immunosuppressive effect may contribute to an immunomodulatory brain cytoprotective approach (Hug et al., 2009; Fugate et al., 2014; Nogueira et al., 2018). Nonetheless, the time window could be better defined and questions about the necessary treatment duration answered. Hence, this study demonstrated a reduction in damaged neurons in a wider and earlier time window of 8–36 h after injury, but not in a time window of 8–24 h. These results led to two conclusions: (i) Starting MMF treatment should occur in the early phase within the first 8 h of injury in order to achieve better results. (ii) Treatment should last longer than 24 h. In agreement, selected patients underwent thrombectomy had a benefit from the therapy when started at 6–24 h (Nogueira et al., 2018; Lyden et al., 2021). Furthermore, after a short treatment a plasma concentration of approximately 100 μg/ml period is certainly achievable without a risk of general immunosuppressive effects as observed after prolonged MMF therapy. Due to fluctuating plasma concentrations of MMF, area under the curve (AUC) was established as a relevant parameter for bioavailability and therapeutic drug monitoring. The ideal AUC has been reported to be 40–60 μg × h/mL (Ferreira et al., 2020) after renal transplantation and about 30–40 μg × h/mL after lung transplantation (Yabuki et al., 2020). However, it should be noted that the bioavailability in these studies was chosen to establish sufficient immunosuppression with few side effects. These concentrations are therefore slightly below the 100 μg/ml used here, although those were collected in the context of a therapy existing over a longer period of time. The therapeutic use of MMF described here refers to a much shorter period of time and is not intended to cause a permanent immune suppression. A short therapeutic period with a higher AUC for immunomodulation would therefore certainly be conceivable. Furthermore, the increasingly established use of interventional therapies such as mechanical thrombectomies (Nogueira et al., 2018) offers the possibility to apply drugs exactly at the desired site and thus to achieve significantly higher concentrations without systematic effect. Taken together, the here used MMF concentration was chosen based on a realistic scenario and represents an achievable tissue concentration also *in vivo*. Possible limitations might be the fluctuating plasma levels, a more complex pharmacokinetics due to unclear perfusion conditions in the penumbra, a changing blood brain barrier and an increasing isolation of the lesion by glial cells.

Mycophenolate mofetil treatments for a short interval from 8 to 24 h led to a reduction in microglia number. This observation was consistent with previous results from an *in vitro* study, in which treatment for longer than 4 h significantly reduced the number of microglia (Ebrahimi et al., 2012). Although increased apoptosis by MMF application was observed in microglia cell culture (Dehghani et al., 2003). MMF treatment reduced the proliferation rate of astrocytes and microglia in OHSC without changing the number of apoptotic cells (Ebrahimi et al., 2012). Thus, the reduced number of microglia can be attributed to a reduction of proliferation. Notably, the observed brain cytoprotective effect was not always correlated with a reduced number of microglia. Decreased microglia number in a treatment window of 8–24 h remained without significant brain cytoprotective evidence. Therefore, a reduced number and proliferation of microglia in the early phase after the injury appear as a consequence of MMF treatment, but presumably without causality for the brain cytoprotective effects.

### Microglia Dependency of Mycophenolate Mofetil Mediated Effects

In general, after CNS injury, increased stimulation through so-called pattern-recognition receptors (PRRs) results in “activation” of microglia with subsequent increased proliferation, migration, and chemotaxis, as well as increased release of cytokines (Deczkowska et al., 2018; Jiang et al., 2020; Ferro et al., 2021). The active form of microglia exerted its effects through increased phagocytic activity, secretion of proinflammatory cytokines, NO, proteases, arachidonic acid derivatives, and expression of several receptors (Simon et al., 2017; Subhramanyam et al., 2019; Takeda et al., 2021). MMF was found to influence cytokine release and NO production in microglia and macrophages. However, effects on astrocytes were observed as well (Miljkovic et al., 2002; Dehghani et al., 2010; Xu et al., 2022). Microglia were depleted from OHSC by clodronate to evaluate the impact of these cells on brain cytoprotective effects. Lesion of almost microglia free OHSC significantly exacerbates NMDA induced damage (Kohl et al., 2003). The results are in line with the increasingly accepted position that microglia, next to their neurotoxic properties, possess many cytoprotective attributes. Often the microglia clearance of CNS debris was misinterpreted as “killing” of neuronal cells. However, it is a more essential function for the maintenance of homeostasis (Derecki et al., 2012; Ferro et al., 2021; Xu et al., 2022). Moreover, microglia had the potential to suppress existing inflammation and to stimulate regenerative processes (Rasley et al., 2006; Thored et al., 2009). This point is particularly illustrated by the fact that after complete microglia depletion the brain cytoprotective effect of MMF at 24, 36, and 48 h were lost. Furthermore, a significant reduction in damaged neurons was also observed after 72 h in microglia depleted OHSC, indicating the involvement of additional cell types, mainly astrocytes in MMF related effects. MMF was found to affect cytokine release and proliferation in astrocytes and influence cell migration and scar formation in scratch assay (Miljkovic et al., 2002; Ebrahimi et al., 2012). A direct effect on neurons seems unlikely (Dehghani et al., 2010). Microglia very likely mediate the major impact on brain cytoprotection in an early phase of injury. This pattern of MMF effects consisting of an early microglia-mediated phase and a possible late astrocytic phase completed the findings from previous study (Ebrahimi et al., 2012). Excitingly, a brain cytoprotective effect was already found 24 h after injury and subsequent fixation. However, no protective effect was observed after application of MMF in a time window of 8–24 h only and fixation after 72 h. Interestingly, MMF treatment for a short period might not be sufficient to prevent a renewed progression of inflammation and secondary damage. However, a prolonged treatment with MMF in a time window of 8–36 h led to satisfactory results. Thus, there might be a critical phase in which a sufficiently long MMF application would be necessary to achieve a consistent protective effect. Our results fit well to time-dependent data obtained from a genetic characterization of microglia after TBI in rats with upregulation of chemotactic genes and downregulation of anti-inflammatory genes 2 days after trauma (Izzy et al., 2019). Furthermore, it is consistent with the implied observations on IMPDH2 expression in microglia and astrocytes.

### IMPDH2 in Glia Cells Seem to Be a Main Target of Mycophenolate Mofetil

After acute neuronal injury, microglia and astrocytes began to proliferate. This is a hallmark of gliosis in several CNS pathologies, including stroke and TBI (Iadecola and Anrather, 2011; Jassam et al., 2017). IMPDH is a major rate limiting enzyme involved in the *de novo* purine biosynthesis and directly affects the proliferation of cells (Allison et al., 1993; Pua et al., 2017). MMF inhibited IMPDH due to the replacement of the nicotinamide adenine dinucleotide (NAD) cofactor (Sintchak et al., 1996) and reduced the guanosine pool, which in turn inhibited proliferation (Allison and Eugui, 2000). Inhibition of IMPDH results primarily in depletion of guanosine monophosphate (GMP) and consequently also of intercellular guanosine triphosphate (GTP) and deoxyguanosine triphosphate (dGTP) (Allison et al., 1993; Gu et al., 2003). Guanosine as well as deoxyguanosine in turn show an allosteric function of 5-phosphoribosyl-1-pyrophosphate (PRPP) synthetase and ribonucleotide reductase which in turn affect the entire purine metabolism and thus the replication of DNA and cell cycle. This inhibition of *de novo* synthesis followed by a reduced intracellular concentration of guanosine nucleotides seems to be one of the determining mechanisms behind the reduction of cytokines in microglia and astrocytes and thus to play a role in the observed brain cytoprotective effects. In a combined treatment with guanosine and MMF in the damaged OHSC, the brain cytoprotective effect was abrogated (Ebrahimi et al., 2012). These results are also consistent with reduced proliferation rates, cytokine, and NO production shown in microglia and astrocytes, which where antagonized by guanosine (Dehghani et al., 2010). Sappanone A (SA), a highly selective inhibitor of IMPDH2, led to a reduction in microglia activation and cytokine production (Liao et al., 2017). Furthermore, SA significantly reduced GTP levels in BV2 cells, and the neuroinflammatory effects were attenuated after application of an IMPDH2 siRNA (Liao et al., 2017). The close link between metabolism and immunological cellular responses, proliferation as well as cell differentiation seems to be an important basis to understand the complex inflammatory reactions. MMF was reported to modulate the activities of Myc *via* NDRG1 in endothelial cells (Domhan et al., 2008). This activity was closely linked to the regulation of cell growth, proliferation, and differentiation (Secombe et al., 2004). In a gastric cancer cell line, treatment with MMF led to an alteration of several cyclins and cyclin kinases at the transcriptional level, indicating an influence on the PI3K/AKT/mTOR pathway (Dun et al., 2013). This could explain the changed expression of genes involved in glutaminolysis, nucleotide synthesis and glycolysis in Jurkat T cells by MMF (Fernandez-Ramos et al., 2017; Zhu and Thompson, 2019). Furthermore, a reduction of HIF-1alpha and Myc but not of Akt and mTORC1 was demonstrated and an independent regulation of Myc and HIF-1alpha by MMF was suggested (Fernandez-Ramos et al., 2017). In human CD4 T cells, an alteration of the Akt/mTOR as well as STAT5 signaling pathway was noticed (He et al., 2011). In addition to the reduction of GTP by MMF, a moderate decrease in adenosine triphosphate (ATP) concentration was detected within a short time in T cells, which in turn might activate AMPK as an energetic sensor (Fernandez-Ramos et al., 2017) and conceivably modulate mTORC1 (Saxton and Sabatini, 2017). The relationship between nucleotide metabolism, the cell cycle, in terms of proliferation and the release of neuroinflammatory cytokines in neuronal injury needs to be further characterized. Previously, the expression of IMPDH2 was demonstrated in OHSC (Ebrahimi et al., 2012). Since microglia belong to the monocytic cell lineage, it was likely that IMPDH2 is particularly expressed in these cells (Ginhoux et al., 2010; Ginhoux and Jung, 2014). Hence, in this study, the expression of IMPDH2 in primary microglia and astrocytes was detected in accordance to an earlier report (Liao et al., 2017). LPS stimulation in primary microglia as well as astrocytes did not result in a quantitative increase of IMPDH2 in comparison to the control which was observed after stimulation of lymphocytes (Dayton et al., 1994). However, there was a general increased amount of IMPDH2 in primary microglia after 12 h in culture. Primary astrocytic culture showed an increase in IMPDH2 after 16 h as well as after 24 h. Among others, early proliferation of microglia accompanied by delayed astrocytic proliferation in cell culture might explain this increase. This would also be consistent with the early microglia-mediated effect and the late microglia-independent effect by MMF. As LPS treatment did not lead to any altered expression, IMPDH2 might be regulated in its kinetics or dependent on protein-protein interactions (Jain et al., 2004). Furthermore, the activation of toll like receptor (TLR) 4 misses to alter the IMPDH2 expression (Liao et al., 2017).

## Conclusion

The application of brain cytoprotective therapeutics for a short period of time and early after neuronal damage is a fundamental prerequisite of an immunomodulating approach. MMF led to a reduction of neuronal damage in OHSC when the treatment was started within the first 8 h of injury and lasted for a period of at least 24 h. MMF showed a brain cytoprotective effect at 24, 36, 48, and 72 h after injury. In addition, no protection was detectable at 24, 36, and 48 h after depleting microglia, indicating an early microglia-dependent phase and a late microglia-independent phase for MMF effects. It is very likely that microglia mediate the major impact for MMF brain cytoprotective effects but the role of astrocytes should be considered particularly in the late phase. The IMPDH2 as a target of MMF was detected in primary microglia as well as in astrocytes. Summarizing our results, MMF might be a potential candidate in the treatment of acute lesions of the CNS.

## Data Availability Statement

The datasets presented in this study can be found in online repositories. The names of the repository/repositories and accession number(s) can be found in the article/Supplementary Material.

## Ethics Statement

All animal experiments were performed in accordance with the Policy on Ethics and the Policy on the Use of Animals in Neuroscience Research as indicated in the directive 2010/63/EU of the European Parliament and of the Council of the European Union on the protection of animals used for scientific purposes and were approved by the local authorities for care and use of laboratory animals (State of Saxony-Anhalt, Germany, permission number: I11M18, date: 01.12.2012).

## Author Contributions

FD, UH, and JK conceptualized and designed the study and wrote the manuscript. JK, UH, TH, CG, MS, SL, and BA conducted the research. JK, UH, and TH analyzed the data. All authors provided critical revisions to the manuscript.

## Conflict of Interest

The authors declare that the research was conducted in the absence of any commercial or financial relationships that could be construed as a potential conflict of interest.

## Publisher’s Note

All claims expressed in this article are solely those of the authors and do not necessarily represent those of their affiliated organizations, or those of the publisher, the editors and the reviewers. Any product that may be evaluated in this article, or claim that may be made by its manufacturer, is not guaranteed or endorsed by the publisher.
