# Supplementary material for: Microglia-Dependent and Independent Brain Cytoprotective Effects of Mycophenolate Mofetil During Neuronal Damage
Source: Front Aging Neurosci. 2022 Apr 29;14:863598. doi: 10.3389/fnagi.2022.863598 (PMC9100558; doi:10.3389/fnagi.2022.863598)
Supplement: Supplementary file 2 [file Data_Sheet_2.docx]

Supplementary Material
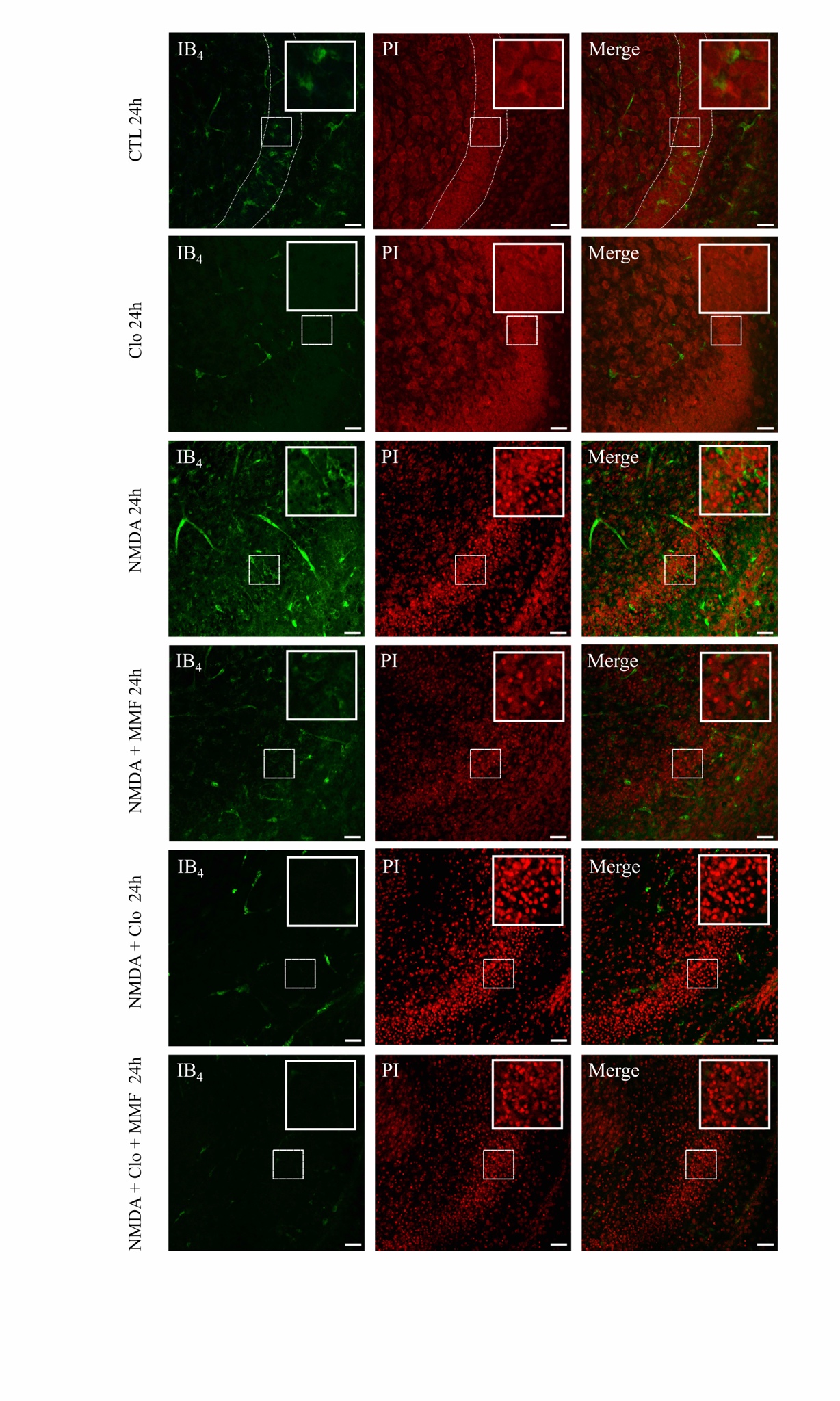


**Supplementary Figure 1.** CLSM images stained with PI (degenerating neurons, red) and IB4 (microglial cells vascular vessels, green) after 24 h. In comparison to controls (CTL), treatment with NMDA for 4h (NMDA) led to an increase in number of PI positive degenerating neurons at all time points. Treatment with MMF (NMDA+MMF) in a period between 4 and 24 h after the injury resulted in a reduction of PI positive degenerated neurons. Incubation with 100µg/ml Clo for 6 days resulted in successful depletion of microglia from OHSC in the respective groups (Clo, NMDA + Clo, NMDA + Clo + MMF). Additional application of MMF led to no significant reduction of PI positive degenerated neurons in microglia depleted OHSC (NMDA + Clo+ MMF). Depletion of microglia led to an increase in number of damaged cells (NMDA + Clo, NMDA + Clo + MMF). The dotted area in the CTL group showed the DG. A 2.5-fold magnification of the respective images was performed. Scale bars = 50 µm.


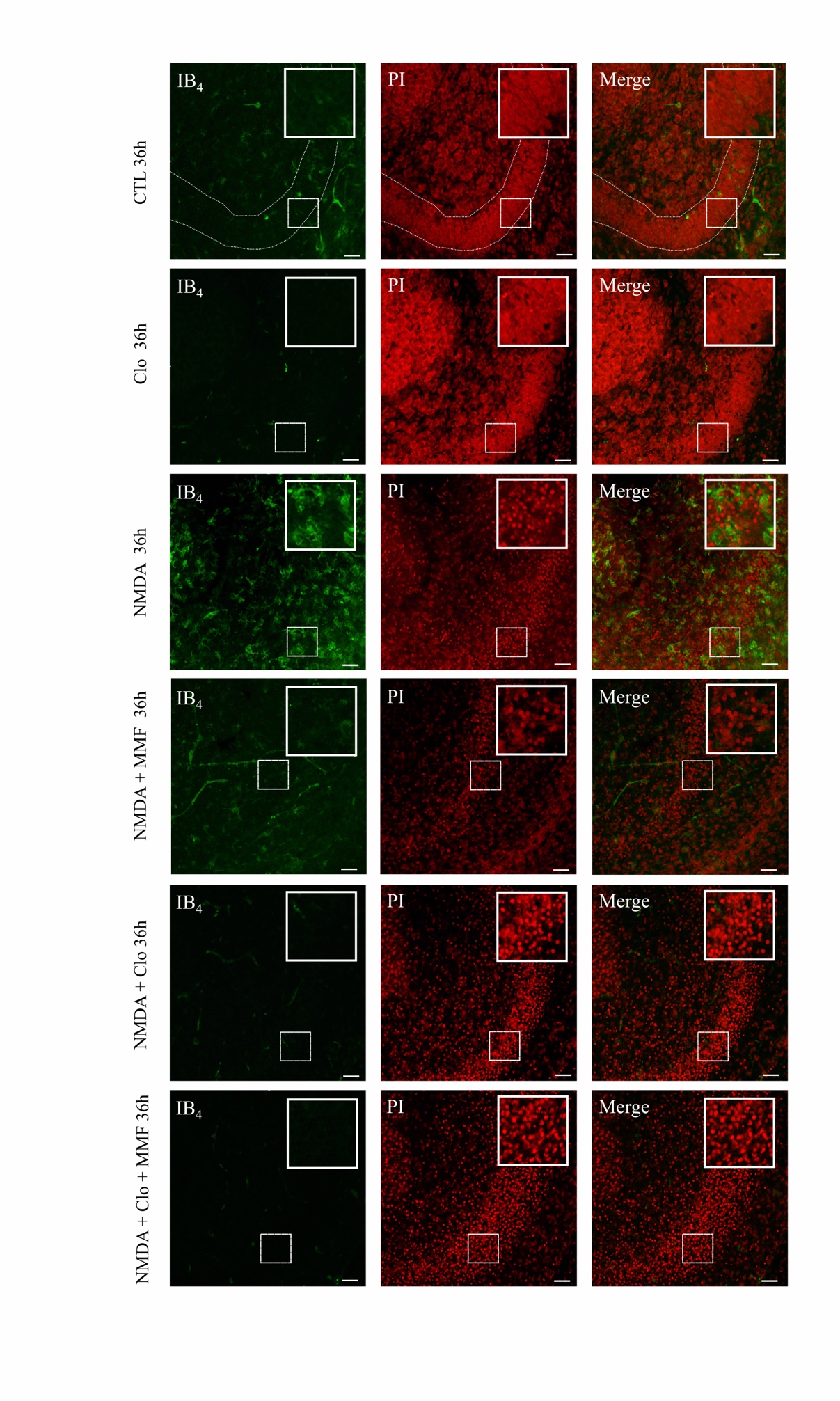


**Supplementary Figure 2.** CLSM images stained with PI (degenerating neurons, red) and IB4 (microglial cells vascular vessels, green) after 36 h. In comparison to controls (CTL), treatment with NMDA for 4h (NMDA) led to an increase in number of PI positive degenerating neurons at all time points. Treatment with MMF (NMDA+MMF) in a period between 4 and 36 h after the injury resulted in a reduction of PI positive degenerated neurons. Incubation with 100µg/ml Clo for 6 days resulted in successful depletion of microglia from OHSC in the respective groups (Clo, NMDA + Clo, NMDA + Clo + MMF). Additional application of MMF led to no significant reduction of PI positive degenerated neurons in microglia depleted OHSC (NMDA + Clo+ MMF). Depletion of microglia led to an increase in number of damaged cells (NMDA + Clo, NMDA + Clo + MMF). The dotted area in the CTL group showed the DG. A 2.5-fold magnification of the respective images was performed. Scale bars = 50 µm.


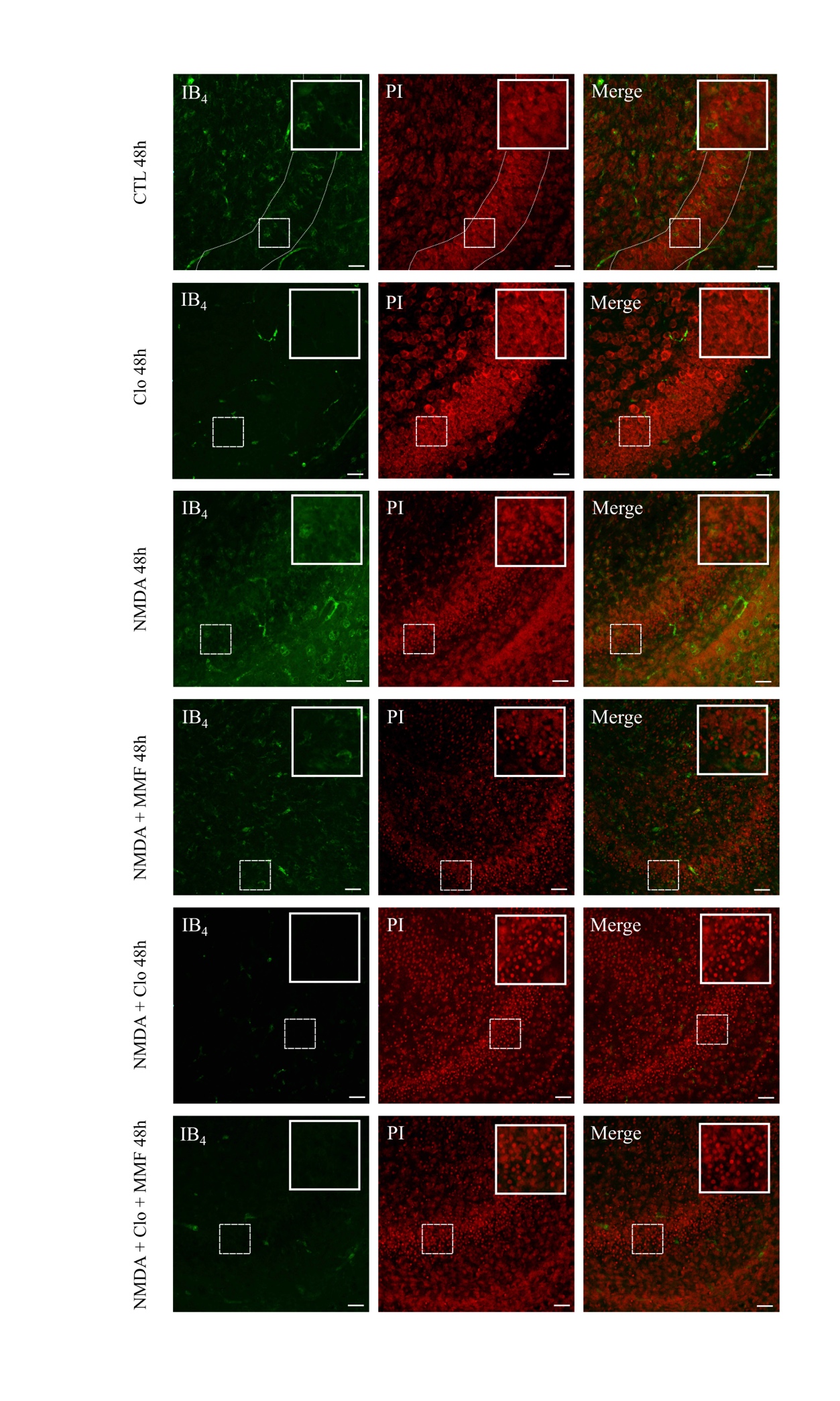


**Supplementary Figure 3.** CLSM images stained with PI (degenerating neurons, red) and IB4 (microglial cells vascular vessels, green) after 48 h. In comparison to controls (CTL), treatment with NMDA for 4h (NMDA) led to an increase in number of PI positive degenerating neurons at all time points. Treatment with MMF (NMDA+MMF) in a period between 4 and 48 h after the injury resulted in a reduction of PI positive degenerated neurons. Incubation with 100µg/ml Clo for 6 days resulted in successful depletion of microglia from OHSC in the respective groups (Clo, NMDA + Clo, NMDA + Clo + MMF). Additional application of MMF led to no significant reduction of PI positive degenerated neurons in microglia depleted OHSC (NMDA + Clo+ MMF). Depletion of microglia led to an increase in number of damaged cells (NMDA + Clo, NMDA + Clo + MMF). The dotted area in the CTL group showed the DG. A 2.5-fold magnification of the respective images was performed. Scale bars = 50 µm.


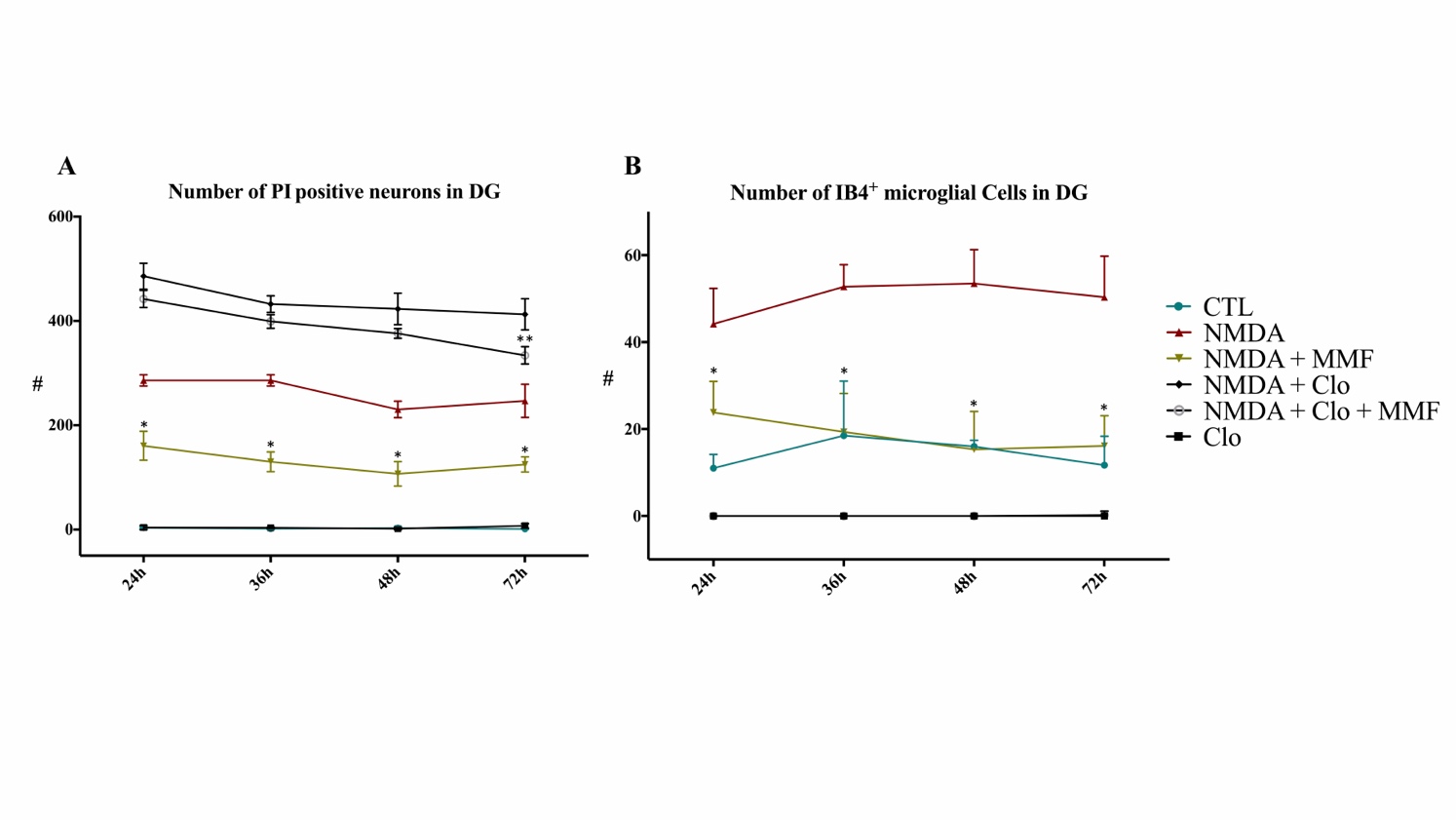


**Supplementary Figure 4.** Effects of continuous MMF over time with and without (Clo) microglia. (**A**) Quantitative analyses of the mean numbers of PI positive degenerating neurons over time (* p<0.05 NMDA + MMF vs. NMDA, ** p<0.05 NMDA + Clo + MMF vs. NMDA + Clo). (**B**) Quantitative analyses of the mean number of IB4 positive microglia over time (* p<0.05 NMDA + MMF vs. NMDA). The asterisk denotes significant results regarding the respective measurement indicated with the bar. The values are served as a mean with standard error of the mean.


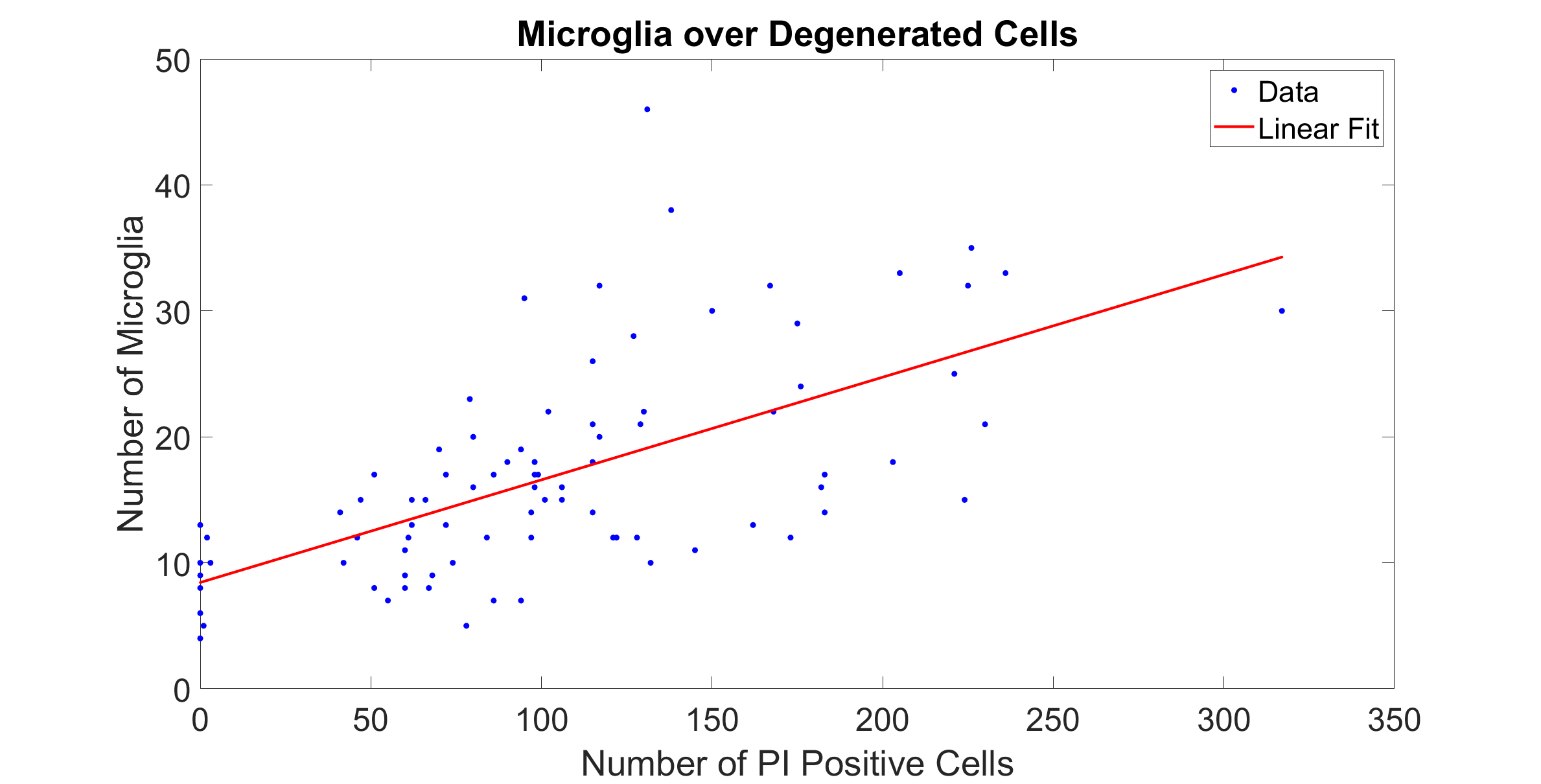


**Supplementary Figure 5.** Number of PI positive plotted against the number of microglia, as well as the linear fit (red). A strong statistically significant correlation was found between the number of PI and IB4 positive cells in all constellations can be observed. A pearson correlation coefficient of 0.631 and a 95% confidence interval of [0.485, 0,743] were observed.


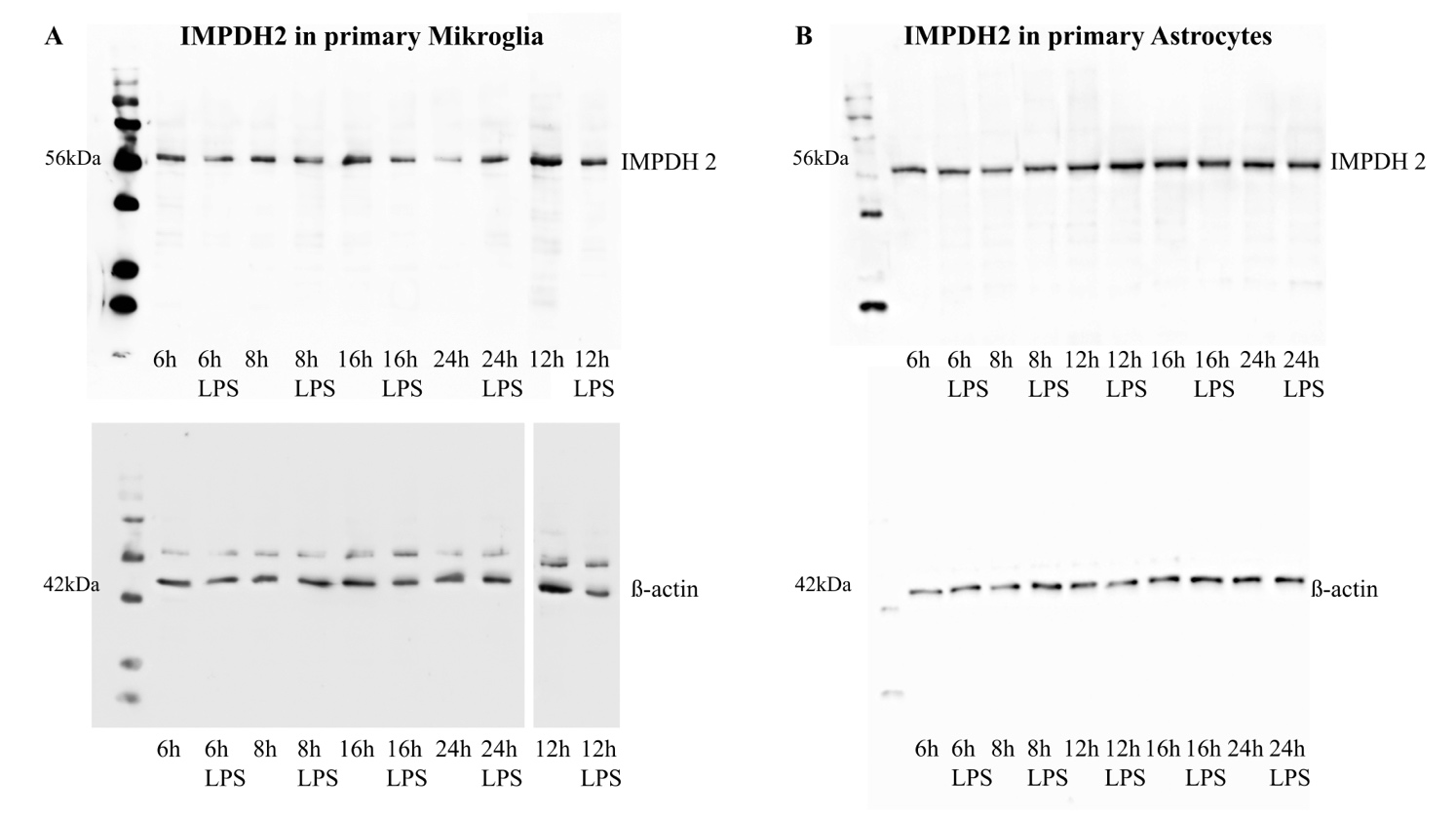


**Supplementary Figure 6.** Immunoblot showing immunoreactivity of the IMPDH2 antibody at 56 kDa and ß-actin at 42 kDa at different time points (0, 6, 8, 12, 16, 24 hours) in (**A**) primary microglia and (**B**) primary astrocytes. LPS showed no effect on IMPDH2 expression in microglia as in astrocytes.
